# Changes in social relationships from 26 to 34 years of age in adults born very preterm

**DOI:** 10.1111/ppe.13133

**Published:** 2024-10-27

**Authors:** Elif Gonen, E Sabrina Twilhaar, Nicole Baumann, Barbara Busch, Peter Bartmann, Dieter Wolke

**Affiliations:** ^1^ Department of Psychology University of Warwick Coventry UK; ^2^ Department of Population Health Sciences University of Leicester Leicester UK; ^3^ Turner Institute for Brain and Mental Health, School of Psychological Sciences Monash University Melbourne Australia; ^4^ Department of Neonatology and Paediatric Intensive Care University Hospital Bonn Bonn Germany; ^5^ Division of Health Sciences, Warwick Medical School University of Warwick Coventry UK

**Keywords:** emerging adulthood, established adulthood, longitudinal study, social relationship characteristics, very low birthweight, very preterm birth

## Abstract

**Background:**

Very preterm and/or very low birthweight (VP/VLBW; <32 weeks' gestation and/or <1500 g birthweight) individuals rated their partner and peer relationships lower than term‐born individuals in emerging adulthood, but their quality of relationships with parents has been rarely investigated. Moreover, it is unclear whether previously reported differences in social relationship characteristics persist or lessen from emerging to established adulthood.

**Objectives:**

To investigate changes in social relationship characteristics in VP/VLBW adults compared to term‐born adults from 26 to 34 years and whether the association between VP/VLBW and social relationship characteristics varies according to sex.

**Methods:**

In this prospective whole‐population birth cohort study in South Bavaria, Germany, social relationship characteristics with parents, partners and peers, and overall social relationships across these domains were evaluated with a Life Course Interview at 26 and 34 years. Interview items related to these domains were extracted and scored as 0 (optimal) and 1 (non‐optimal). Each score was summed into domain‐specific composite scores and standardised according to the total sample.

**Results:**

Participants included 262 VP/VLBW (52.7% males) and 230 term‐born individuals (47.0% males). VP/VLBW adults had lower overall social relationship scores than term‐born adults (*β* = −.61, 95% CI −0.85, −0.37). Specifically, partner (*β* = −.50, 95% CI−0.74, −0.27) and peer relationship scores (*β* = −.55, 95% CI−0.78, −0.32) were lower than those of term‐born adults, but scores did not differ for parent relationships. On average, partner (*β* = .25, 95% CI 0.14, 0.35) and peer relationship scores increased (*β* = .16, 95% CI 0.03, 0.29), while parent relationship scores decreased (*β* = −.64, 95% CI−0.79, −0.49) from 26 to 34 years. These changes were similar for VP/VLBW and term‐born individuals.

**Conclusions:**

Patterns of change for the improved partner and peer but worsening parental social relationship scores were common across VP/VLBW and term‐born adults, but differences between the two groups persisted from 26 to 34 years.

**Synopsis**

**Study Question**

Do differences between very preterm and/or very low birthweight (VP/VLBW) and term‐born adults in social relationship characteristics narrow, persist or widen from emerging to established adulthood?

**What's Already Known**

On average, VP/VLBW young adults have fewer friends than their term‐born peers in emerging adulthood (in their twenties). Moreover, fewer of them experience sexual intercourse, form romantic relationships, get married or cohabit with a partner during this period.

**What this Study Adds**

Differences between VP/VLBW and term‐born adults in partner and peer relationship characteristics remained stable across emerging and established adulthood. The main reason for this difference was initiating relationships, i.e. making friends and finding romantic partners. In contrast, the quality of relationships with parents was comparable between VP/VLBW and term‐born adults.

## 1 BACKGROUND

Having social relationships and the quality of these relationships are both associated with increased self‐esteem, better health and longevity.^1^, ^2^, ^3^ However, on average, individuals born very preterm (VP; <32 weeks' gestation) and/or with very low birthweight (VLBW; <1500 g) have been reported to have fewer friends, lower peer acceptance^4^, ^5^ and are more often bullied in childhood than term‐born children (≥37 weeks' gestation).^6^ In emerging adulthood (ages 18–29), peer relationship characteristics are still rated lower more often by VP/VLBW individuals than term‐born individuals^7^ with little change in trajectories from childhood.^8^ Moreover, research has suggested that fewer VP/VLBW adults, on average, experienced sexual intercourse, formed romantic relationships, got married or cohabited with a partner compared to term‐born adults during emerging adulthood,^7^, ^9^, ^10^ but the quality of their relationship is similar once they have a partner.^7^, ^10^ Their relationships with parents in adulthood have rarely been investigated. One previous study found stronger parent relationships in VP/VLBW adults than in term‐born adults.^11^ In contrast, the social bonds of VP/VLBW individuals with their parents may be less strong compared to their term‐born peers, given previously reported concerns about the effects of the separation during incubator care on parenting.^12^

In high‐income countries, established adulthood (ages 30–45) is a defining and demanding period of life with many changes, including family formation and increasing career responsibilities that may alter social relationships forged in emerging adulthood.^13^ However, there is a paucity of repeated longitudinal assessments of VP/VLBW social relationships beyond emerging adulthood.^10^ It is thus unknown whether differences in social relationship characteristics persist, decrease,could not or even increase into established adulthood. Furthermore, it is unclear whether social relationship characteristics of VP/VLBW adults differ by sex, although no differences between VP/VLBW men and women have been found in emerging adulthood.^7^

The current study investigated differences between VP/VLBW and term‐born adults in social relationship characteristics with parents, partners and peers and changes in these differences from emerging (26 years) to established adulthood (34 years). We tested two alternative hypotheses: (1) differences in social relationship characteristics between VP/VLBW and term‐born adults lessen into established adulthood; (2) they remain the same or increase by 34 years. Furthermore, it was tested whether the effect of VP/VLBW on social relationship characteristics differs between males and females.

## 2 METHODS

### 2.1 Cohort selection

**FIGURE 1:**
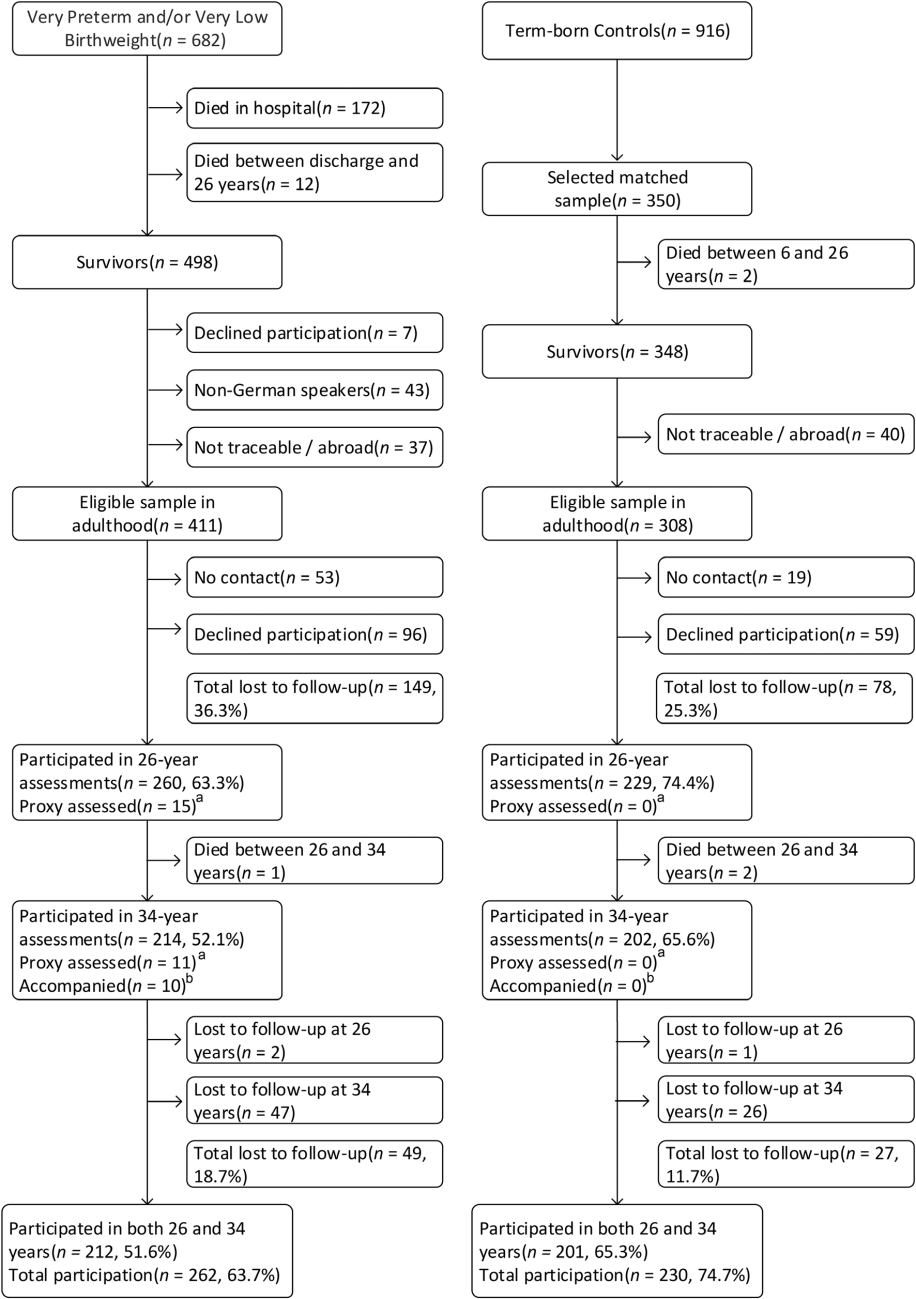
Flow chart describing eligible sample and lost to follow‐up in VP/VLBW and term‐born participants at 26 and 34 years of age. ^a^Parents or caregivers as proxy informants provided information on behalf of participants. ^b^Participants were accompanied by their parents or caregivers during the interview.

The Bavarian Longitudinal Study (BLS) is a geographically defined prospective whole‐population sample of children born in South Bavaria (Germany) between January 1985 and March 1986 who required admission to one of 17 paediatric hospitals within the first 10 days after birth (*N* = 7505; 10.6% of all live births).^14^ Of these, 682 were born VP/VLBW, 411 were alive and eligible for the 26‐year assessment and 260 (63.3%) participated.^15^ At 34 years, 214 (52.1%) VP/VLBW adults participated (Figure 1).

Healthy infants born at term in the same hospitals were recruited as controls. Of the initial 916 children alive at 6 years, 350 were randomly selected as term controls within the stratification variables sex and family socioeconomic status (SES) to be comparable to the VP/VLBW sample. Of these, 308 were eligible for the 26‐year assessment, 229 (74.4%) participated at 26 years,^15^ and 202 (65.6%) participated at 34 years (Figure 1).

In total, 262 VP/VLBW (52.7% males) and 230 term‐born (47.0% males) individuals were assessed at 26 and/or 34 years. No data were available for one of the two assessments for 50 VP/VLBW (19.1%) and 29 term‐born (12.6%) individuals. For 15 VP/VLBW adults who were unable to complete the interview by themselves at either time, parents or caregivers served as proxy informants. At 34 years, 10 VP/VLBW individuals needed assistance by parents or caregivers during the interviews.

### 2.2 Outcome

Information on social relationship characteristics was obtained with a standard Life Course Interview (LCI) at 26 and 34 years. The LCI was developed for the BLS based on items of several established and widely used life course instruments from the German Socioeconomic Panel Study^16^, ^17^ and the Avon Longitudinal Study of Parents and Children.^18^ At 26 years, 399 of the interviews (81.6%) were conducted face‐to‐face. Additionally, to maximise participation, 50 telephone (10.2%) and 15 proxy interviews (3.1%) were conducted, and 25 of the interviewees (5.1%) completed the interviews via questionnaires. At 34 years, interviews were carried out in the context of a telephone interview.

Interview items related to different characteristics of parent, partner and peer relationships were extracted from the LCI. Each item was dichotomised and scored as ‘0’ indicating optimal and ‘1’ indicating non‐optimal scores. There were six dichotomised items concerning parent relationship characteristics (scores based on the quality of parent relationships; e.g. ‘stressful conflicts with parents’), seven items regarding partner relationship characteristics^19^ (based on romantic/sexual experience and violence in the relationships; e.g. ‘never dated’) and ten items concerning peer relationship characteristics^8^ (based on having friends, the quality of peer relationships and socialising; e.g. ‘no best friend after school’). Item scores of each domain were summed into domain‐specific composite scores, and an overall social relationship score was computed by summing these scores (eTable S1). Higher scores thus indicated a higher frequency of non‐optimal scores. For ease of interpretation, scores were standardised according to the total sample and reverse coded with higher scores indicating a higher frequency of optimal scores. Similar composite scores totalling across items measuring social relationships in different samples have been validated and published previously.^20^, ^21^

In total, 51 interviews had structurally missing data^22^ in individual items from the proxy, accompanied or partial assessments due to the lack of initial experience (e.g. missing data on experiencing violence in romantic relationships if hadn't had a romantic relationship before). Using a conservative approach, structurally missing data were assigned a score of 1 (non‐optimal) only when certain situations sensibly suggested an adverse effect on the relevant item. Otherwise, they were recoded as 0 (optimal), which was the same as leaving them missing. For instance, missing data for the item ‘No exchange of thoughts and feelings with friends’ was recoded to score 1 (non‐optimal) if they had no friends to share thoughts and feelings with (Data S1). Additionally, sum scores for each domain were recalculated without this missing data substitution (i.e. leaving them missing), and a sensitivity analysis was conducted.

Forty LCI interviews were audio‐recorded and independently rated by two raters for interrater reliability.^23^ Intraclass correlation coefficients were: .94 for overall social, .97 for parent, .97 for partner and .96 for peer relationships.

### 2.3 Descriptive variables

Gestational age (completed weeks), birthweight and biological sex were extracted from birth records.^24^ Small for gestational age was defined as birthweight below the sex‐specific 10th percentile for gestational age.^25^ Family SES data were obtained by standard interviews with the infants' parents in the first 10 days of life. SES was computed as a weighted composite score of maternal highest educational qualification, paternal highest educational qualification and occupation of the head of family, and was classified into three categories as low, middle or high.^24^, ^25^ Bronchopulmonary dysplasia and intraventricular haemorrhage were diagnosed in the neonatal period.^26^ At 6 and 8 years, intelligence was assessed with the German version of the Kaufman Assessment Battery for Children Mental Processing Composite,^27^, ^28^ and neuro‐sensory impairments (NSI) were defined as severe cerebral palsy (grade 3 or 4),^29^ hearing loss (uncorrected), blindness or IQ more than 2 SD below the mean^24^ (Data S2).

### 2.4 Statistical analyses

The study was preregistered on the Open Science Framework on 1st June 2023 (https://osf.io/s4pzm). Analyses were conducted using SPSS Version 29 (IBM SPSS Statistics, IBM Corporation).

The main effects of birth group (VP/VLBW, term‐born) and age (26, 34 years) and their interaction effect (birth group*age) on social relationship characteristics were tested to investigate differences between VP/VLBW and term‐born adults, changes from 26 to 34 years and differences in this change between VP/VLBW and term‐born individuals, respectively. Furthermore, the main effect of sex (males, females) and the interaction effect (birth group*sex) were tested to investigate sex differences in social relationship characteristics and in the relation between VP/VLBW and social relationship characteristics, respectively. Linear mixed model (LMM) analysis was used to investigate each effect on social relationship domains (overall social, parent, partner, peers), considering age as a within‐subject factor and birth group and sex as between‐subject factors. Effect sizes (*β*) between 0.10 and 0.29 were considered small, 0.30 and 0.49 medium, and ≥0.50 large.^30^

All available data (*n* = 492) were included in the LMM analysis without multiple imputations.^31^ Model parameters were estimated with a restricted maximum likelihood procedure. A random intercept was included in all models to adjust for repeated measurements within individuals. Random slopes were considered to allow the change in social relationship scores to vary between individuals. A likelihood ratio test was conducted to examine whether the inclusion of random slopes in addition to the random intercept provided a better model fit.^32^

### 2.5 Missing data

At 26‐ and/or 34‐year assessments, 149 of the VP/VLBW (36.3%) and 78 term‐born participants (25.3%) were eligible to participate but lost to follow‐up. As loss to follow‐up was related to family SES at birth in both groups and birthweight in the VP/VLBW group, the inverse probability of censoring weighting (IPCW) was implemented before the primary analyses to account for selective dropout (in addition to the preregistration, IPCW was conducted as a sensitivity analysis). The weights were calculated based on a logistic regression model according to the inverse probability of participating in the study. Birth group, family SES, and birthweight were used as predictors in the model. The weights were not stabilised, as no extreme values were encountered.

### 2.6 Sensitivity analyses

Sensitivity analyses were conducted by repeating LMM analysis without recoding missing items from 51 assessments, excluding participants with NSI (*n* = 43) and after implementing IPCW.

### 2.7 Ethics approval

Ethical approval at birth was obtained from the University of Munich Children's Hospital and the Bavarian Health Council (Landesärztekammer Bayern), and for the 26‐year (#159/09) and 34‐year assessments (#281/18) by the Ethical Board of the University Hospital Bonn. Informed written consent was provided initially by parents within 48 hours of birth and all participants gave fully informed written consent for the assessments in adulthood. In case of severe impairment of the adult participant, consent was provided by an assigned guardian (usually a parent).^33^

## 3 RESULTS

### 3.1 Sample demographics and dropout analysis

At birth, VP/VLBW individuals, by definition, had lower birthweight and gestational age and were more often small for gestational age than term‐born individuals. They experienced more often bronchopulmonary dysplasia and intraventricular haemorrhage and were more often born to families with lower SES than term‐born peers. The frequency of male sex was similar in the two groups. In childhood, NSI was more frequent in VP/VLBW individuals (Table 1). Individuals who were eligible for participation but not assessed in adulthood (i.e. lost to follow‐up) were compared to participants assessed at least once in adulthood on the same descriptive characteristics. Loss to follow‐up was associated with lower SES and lower birthweights in the VP/VLBW group and with lower SES in the term‐born group (eTable S2). Among those who participated in the 26‐ and/or 34‐year assessments, missing one assessment (*n* = 79; Figure 1) was associated with lower family SES in the VP/VLBW sample and with male sex in the term‐born sample (Table 1).

**TABLE 1:** Descriptive characteristics of VP/VLBW and term‐born participants.

Descriptive characteristics	VP/VLBW			Term‐born			VP/VLBW versus term‐born
	Data available at both time points *n* = 212 (80.9%)	Data available at one time point *n* = 50 (19.1%)	Difference (95% CI)	Data available at both time points *N* = 201 (87.4%)	Data available at one time point *N* = 29 (12.6%)	Difference (95% CI)	Difference (95% CI)
Birthweight, mean (SD), g	1313 (311.7)	1377 (334.9)	−64.1 (−162.0, 33.8)	3363 (459.6)	3346 (335.8)	17.9 (−156.8, 192.5)	−2036.1 (−2103.9, −1968.2)
Gestational age, mean (SD), week	30.5 (2.1)	31.1 (2.6)	−0.57 (−1.3, 0.1)	39.7 (1.2)	39.6 (1.1)	0.1 (−0.4, 0.5)	−9.0 (−9.4, −8.7)
SGA (<10%), No. (%)	86 (40.6)	23 (46.0)	0.8 (0.4, 1.5)	20 (10.0)	3 (10.3)	0.96 (0.3, 3.4)	6.4 (3.9, 10.5)
Sex, No. (%)							
Male	110 (51.9)	28 (56.0)	0.8 (0.5, 1.6)	88 (43.8)	20 (69.0)	0.4 (0.2, 0.8)	1.3 (0.9, 1.8)
Family SES at birth, No. (%)							
SES‐high	48 (22.6)	7 (14.0)	1.00 (Reference)	69 (34.3)	8 (27.6)	1.00 (Reference)	1.00 (Reference)
SES‐middle	104 (49.1)	19 (38.0)	1.6 (0.8, 3.0)	87 (43.3)	11 (37.9)	1.2 (0.6, 2.8)	1.2 (0.8, 1.7)
SES‐low	60 (28.3)	24 (48.0)	0.4 (0.2, 0.8)	45 (22.4)	10 (34.5)	0.5 (0.2, 1.3)	1.5 (1.01, 2.2)
Bronchopulmonary dysplasia, No. (%)	109 (51.4)	28 (56.0)	0.8 (0.4, 1.5)	NA	NA	NA	NA
Intraventricular haemorrhage, No. (%)							
Stage 3 or 4	15 (7.1)	5 (10.0)	0.7 (0.2, 2.0)	0 (0.0)	0 (0.0)	NA	NA
Neurosensory impairments, No. (%)							
One or more imp.	33 (15.6)	9 (18.0)	0.8 (0.4, 1.9)	1 (0.5)	0 (0.0)	NA	43.7 (6.0, 320.4)

*Note*: Independent samples *t*‐test for continuous variables, and $X^{2}$ test or Fisher's exact test for categorical variables were performed. Mean differences for continuous variables and odds ratios for categorical variables were reported, respectively.

Abbreviation: SD, standard deviation; SES, socioeconomic status; SGA, small for gestational age; NA, not available

### 3.2 Social relationship characteristics of VP/VLBW and term‐born individuals

**FIGURE 2:**
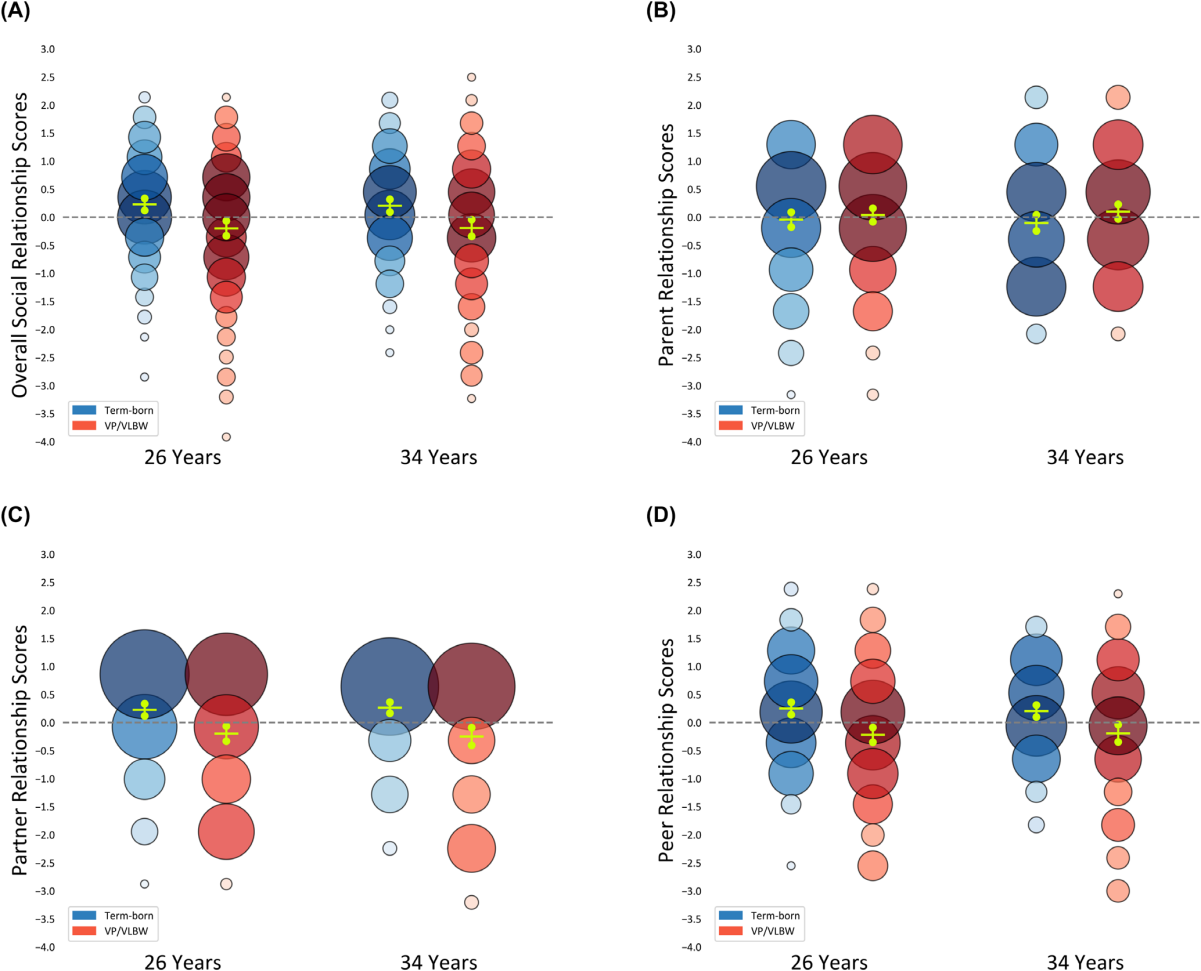
Standardised scores per domain (A, overall social relationships; B, parent relationships; C, partner relationships; D, peer relationships, respectively) and birth group (VP/VLBW, term‐born) with 95% CIs. The increased size and darker shading of circles represent more frequent scores.

Figure 2 shows the distribution of standardised social relationship scores per domain and birth group. The distribution of the scores showed that the majority of the VP/VLBW adults overlapped in their scores with the term‐born adults, but lower relationship scores were more frequent in the VP/VLBW group compared to the term‐born group (Figure 2). On average, across 26 and 34 years, VP/VLBW had a large negative effect on overall social (*β* = −0.61, 95% CI −0.85, −0.37), partner (*β* = −0.50, 95% CI −0.74, −0.27) and peer relationship scores (*β* = −0.55, 95% CI −0.78, −0.32). Looking at the individual items, the largest differences between birth groups were in initially finding friends or partners and participating in social activities (eTable S1). There was no difference between VP/VLBW and term‐born adults in parent relationship scores (Table 2).

**TABLE 2:** Model results for main analysis per social relationship domain and sensitivity analyses.

	Social Relationships β (95% CI)	Sensitivity Analysis‐SMD^a^ β (95% CI)	Sensitivity Analysis‐Excluding NSI^b^ β (95% CI)	Sensitivity Anaysis‐IPCW^c^ β (95% CI)
Overall social relationships				
Birth group	−0.61 (−0.85, −0.37)	−0.58 (−0.82, −0.34)	−0.42 (−0.66, −0.19)	−0.61 (−0.85, −0.37)
Age	−0.11 (−0.24, 0.01)	−0.12 (−0.25, 0.02)	−0.11 (−0.24, 0.01)	−0.11 (−0.23, −0.01)
Sex	−0.07 (−0.30, 0.15)	−0.07 (−0.30, 0.15)	−0.07 (−0.28, 0.14)	−0.07 (−0.29, 0.15)
Birth group*age	0.10 (−0.07, 0.28)	0.17 (−0.02, 0.35)	0.11 (−0.07, 0.29)	0.10 (−0.07, 0.28)
Birth group*sex	0.29 (−0.02, 0.60)	0.31 (0.003, 0.62)	0.27 (−0.03, 0.56)	0.29 (−0.02, 0.60)
Parent relationships				
Birth group	−0.06 (−0.28, 0.17)	−0.04 (−0.25, 0.18)	−0.01 (−0.25, 0.23)	−0.06 (−0.28, 0.17)
Age	−0.64 (−0.79, −0.49)	−0.82 (−0.95, −0.69)	−0.65 (−0.80, −0.50)	−0.64 (−0.79, −0.49)
Sex	−0.23 (−0.44, −0.03)	−0.18 (−0.39, 0.02)	−0.22 (−0.43, −0.02)	−0.23 (−0.43, −0.03)
Birth group*age	0.10 (−0.11, 0.31)	0.14 (−0.04, 0.33)	−0.03 (−0.24, 0.19)	0.09 (−0.11, 0.30)
Birth group*sex	0.29 (0.01, 0.57)^d^	0.28 (0.00, 0.57)	0.31 (0.01, 0.60)	0.29 (0.01, 0.57)
Partner relationships				
Birth group	−0.50 (−0.74, −0.27)	−0.48 (−0.72, −0.25)	−0.33 (−0.57, −0.10)	−0.51 (−0.74, −0.28)
Age	0.25 (0.14, 0.35)	0.25 (0.14, 0.36)	0.25 (0.15, 0.35)	0.23 (0.13, 0.34)
Sex	0.02 (−0.22, 0.25)	0.02 (−0.21, 0.25)	0.02 (−0.20, 0.23)	0.01 (−0.21, 0.24)
Birth group*age	−0.05 (−0.20, 0.10)	−0.01 (−0.17, 0.14)	0.00 (−0.15, 0.15)	−0.04 (−0.19, 0.11)
Birth group*sex	0.12 (−0.20, 0.44)	0.11 (−0.21, 0.42)	0.06 (−0.25, 0.37)	0.12 (−0.19, 0.44)
Peer relationships				
Birth group	−0.55 (−0.78, −0.32)	−0.52 (−0.76, −0.29)	−0.42 (−0.65, −0.19)	−0.56 (−0.78, −0.33)
Age	0.16 (0.03, 0.29)	0.17 (0.03, 0.30)	0.16 (0.04, 0.29)	0.16 (0.04, 0.28)
Sex	0.07 (−0.15, 0.30)	0.08 (−0.15, 0.30)	0.06 (−0.14, 0.27)	0.07 (−0.15, 0.29)
Birth group*age	0.12 (−0.06, 0.30)	0.16 (−0.03, 0.34)	0.18 (0.0004, 0.36)	0.11 (−0.07, 0.29)
Birth group*sex	0.12 (−0.19, 0.43)	0.14 (−0.16, 0.45)	0.12 (−0.17, 0.42)	0.13 (−0.18, 0.43)

*Note*: Birth group, term‐born = 0.00 (Reference); age, 26 years = 0.00 (Reference); sex, female = 0.00 (Reference).

Abbreviations: CI, confidence Interval; IPCW, inverse probability of censoring weighting; NSI, neurosensory impairments; SMD, structurally missing data.

^a^ Linear mixed model analysis without structurally missing data substitution.

^b^ Linear mixed model analysis excluding the participants with neurosensory impairments.

^c^ Linear mixed model analysis after implementing inverse probability of censoring weighting.

^d^ Term‐born males had the lowest parent relationship scores. Standardised mean relationship scores per group: VP/VLBW males M = 0.1 (SD = 0.9) and M = 0.1 (SD = 1.0); VP/VLBW females M = −0.02 (SD = 1.1) and M = 0.1 (SD = 1.0); term‐born males M = −0.1 (SD = 1.0) and M = −0.3 (SD = 0.9); term‐born females M = 0.03 (SD = 1.0) and M = 0.04 (SD = 1.1) at 26 and 34 years, respectively.

### 3.3 Changes in Social Relationship Characteristics

Overall, social relationship scores did not change from 26 to 34 years and there was no interaction of birth group and age. Looking at specific domains, parent relationship scores decrease by established adulthood, with a large effect size (*β* = −0.64, 95% CI −0.79, −0.49). Moreover, small increases in romantic (*β* = 0.25, 95% CI 0.14, 0.35) and peer relationship scores (*β* = 0.16, 95% CI 0.03, 0.29) were observed from 26 to 34 years. None of these effects differed between VP/VLBW and term‐born individuals (Table 2). Random slopes, compared to fixed slopes, provided a better model fit for overall social and parent relationship domains, indicating that the change from 26 to 34 years varied between individuals (Data S3).

### 3.4 Sex differences in social relationship characteristics

The association between VP/VLBW and term‐born individuals with parent relationship scores varied according to sex, showing a stronger effect in males with VP/VLBW males scoring parent relationships higher than term‐born males (interaction: *β* = .29, 95% CI 0.01, 0.57; Table 2). Except for this interaction, no other effect of sex was found. eTable S3 compares males and females on the individual items per birth group.

### 3.5 Sensitivity analyses

The exclusions of recoded missing items from 51 interviews and the participants with NSI (*n* = 43) and implementing IPCW resulted in similar findings (Table 2). However, they affected the individual variation in the change in social relationship characteristics from 26 to 34 years for some domains (Data S3).

## 4 COMMENT

### 4.1 Principal findings

This longitudinal study investigated whether differences in social relationship characteristics between VP/VLBW and term‐born adults change into established adulthood. Our results support the hypothesis that differences between the two groups persisted to 34 years and did not lessen. On average, VP/VLBW adults rated their partner and peer relationships persistently lower than term‐born individuals across 26 and 34 years, and thus a persistent gap in overall social relationship characteristics was found. However, they had similar quality of relationships with their parents as term‐born adults. Despite persistent differences in partner and peer relationships between the groups, VP/VLBW adults followed a similar change pattern in the social relationship characteristics with their term‐born peers from emerging to established adulthood. On average, across the groups, partner and peer relationship scores increased whereas parent relationship scores decreased by 34 years.

### 4.2 Strengths of the study

It is a prospective population‐based birth cohort longitudinal follow‐up study with a large sample for sufficient statistical power. The term‐born comparison group was recruited in the same hospitals and in the same years to diminish any potential selection biases and were matched by sex and family SES to be comparable to the VP/VLBW group. The assessment was based on several established and widely used life course instruments. Moreover, this study reports on different types of relationship characteristics including parent, partner and peer relationships that allowed the evaluation of different aspects of social life.

### 4.3 Limitations of the data

Dropout occurred but response rates were still high with 63% for VP/VLBW and 74% for term‐born adults after 26 years. The loss to follow‐up was higher among participants from lower SES families and VP/VLBW of lower birthweight, as also reported in other samples.^34^ Also, term‐born adult participants were from higher SES families compared to VP/VLBW participants. However, the sample was weighted by the inverse of the probability of participation in the study to account for the potential selection bias, and this implementation resulted in very similar results in the analysis. The reports on their relationships are by those with lived experience, the VP/VLBW adults. We consider this as a strength. However, future studies may want to look at other informants such as parents, partners or friends for a wider perspective. Furthermore, biological sex at birth was used to assess sex differences in social relationship characteristics instead of gender due to the limitation of the data. Lastly, since the study region was limited to South Bavaria, Germany, cultural differences in social relationships may affect the generalisability of results to other regions.

### 4.4 Interpretation

The persistence of differences in peer relationships between preterm and term‐born individuals from childhood to emerging adulthood has been previously reported.^8^ Similar differences have been reported for other developmental domains as well such as growth, cognitive development and attention.^33^, ^35^, ^36^ This study adds that there is no narrowing of the gap from emerging to established adulthood in the characteristics of social relationships with partners and peers and thus no evidence that previously reported differences lessen with advancing adulthood.

The results are consistent with existing meta‐analyses showing that during emerging adulthood, VP/VLBW individuals have, on average, less often romantic relationships and rate their peer relationships lower.^7^, ^10^ Having friends and being liked by them are associated with finding and maintaining romantic relationships.^37^ Notably, in our study, the main reason for the difference between VP/VLBW and term‐born individuals was related to initiating relationships, i.e. finding friends and partners. Fewer VP/VLBW adults engage in activities such as going out where they may meet other adults and potential partners (eTable S1). However, for those who have friends and partners, the quality of the relationships was not different from those of term‐born adults.^7^, ^10^ This suggests that a higher proportion of VP/VLBW adults may be more inhibited to seek and initiate social interactions with peers that are unknown to them. Personality differences after preterm births such as being more introverted^38^ and more likely to be shy, inhibited and withdrawn^39^, ^40^ have been previously reported. Furthermore, more VP/VLBW individuals meet the diagnostic criteria for autism spectrum disorder compared to their term‐ born peers.^41^ These differences may have a neurological basis associated with preterm birth such as the brain being immature and additionally superimposed complications that require brain reorganisation.^42^ For example, a more withdrawn personality is related to differences in brain activation,^43^ and recent meta‐analyses and systematic reviews have summarised the wide‐ranging structural and functional brain alterations in VP/VLBW individuals that are still detectable in adulthood.^44^, ^45^ In VP/VLBW adolescents, alterations in brain areas involved in emotional processing and impairments in cognitive control have been found to be associated with difficulties in social relationships.^46^, ^47^

Children who are socially withdrawn and timid often have parents who may try to compensate by being protective.^48^ Indeed, parents of VP/VLBW children more often consider their offspring as vulnerable^49^ and are overprotective in their parenting.^50^ This overcontrolling behaviour may reduce social encounters or increase social anxiety^51^ and is associated with a higher risk of peer victimisation.^52^ Indeed, VP/VLBW children have been reported to be more often victims of peer bullying.^6^ Peer bullying adversely affects trust in others and social relationships in adolescence and adulthood.^20^ Overprotective parenting and difficulties with peers have been shown to increase anxiety about negative social evaluation, leading to shyness in social interactions.^53^ Thus, this interplay between personality, overprotective parenting and peer victimisation during childhood may increase the proportion of shy VP/VLBW individuals that find it difficult to make initial social contacts.

The quality of parent relationships was not different in VP/VLBW than in term‐born adults across 26 and 34 years. This is reassuring considering the concern that separation due to incubator care and restricted visiting hours practised in many NICU's in the 1980s may have long‐term effects on the relationships of VP/VLBW children and parents.^12^ Our results for VP/VLBW adults and elsewhere reported for parents^54^ indicate that from both perspectives, the parent–child relationship appears to be remarkably resilient to disruption during the neonatal period. The only previous study investigating parent relationships even found closer relationships in VLBW adults than in term‐born adults.^11^ Higher ratings in the quality of parent relationships into adulthood may be maintained as VP/VLBW individuals leave their parents' homes later and require longer financial support in countries where young adults are not supported by universal benefits.^55^

The difference between VP/VLBW and term‐born adults in romantic and peer relationship characteristics was similar in males and females. The only sex difference that was found revealed that term‐born males had the lowest ratings in parent relationship characteristics across sex and birth groups. Previous research indicated that parents' support may be stronger for adult‐daughters^56^ and adult‐children in need^57^ which may include VP/VLBW adult‐offspring.

Finally, our results show that social transitions from emerging to established adulthood are accompanied by improved peer and romantic relationship scores but lessened parent relationship scores for both VP/VLBW and term‐born adults. Compared to emerging adulthood, more adults get married, have children and gain financial autonomy in established adulthood in high‐income countries.^13^ Considering the increased commitment to intimate relationships and careers during this period,^13^ the support previously provided by parents may be replaced by spouses^56^ and by increased financial independence.^58^ Indeed, the decline in parent relationships was accompanied by improved partner and peer relationships in both VP/VLBW and term‐born adults. These improvements may be explained by the socioemotional selectivity theory.^59^ With age, individuals focus on improving relationship satisfaction, gain social expertise and regulate their emotions better,^60^ which may contribute to increased relationship quality with partners and peers.

## 5 CONCLUSIONS

Our results show that the majority of VP/VLBW adults rated their relationships similar to term‐born individuals in adulthood. However, on average, there was a persistent group difference in partner and peer relationship characteristics across 26 and 34 years, with no evidence of lessening over time. The reason for this difference appears to be related to making initial contact with peers and potential partners rather than the relationship quality. We may consider new methods of facilitating new social contacts in adulthood ranging from support groups for adult VP/VLBW or dating applications tailored to people who are inhibited to make initial contacts. Furthermore, identifying early risk and protective factors related to social relationships of VP/VLBW individuals, and endeavouring to strengthen social connections in childhood may have a long‐term positive impact.

## AUTHOR CONTRIBUTIONS

Elif Gonen designed the study, analysed and interpreted the data, drafted and critically reviewed and revised the manuscript. E Sabrina Twilhaar contributed to the conceptualisation and design of the study, analysed and interpreted the data, and critically reviewed and revised the manuscript. Nicole Baumann managed the data obtained, carried out the initial analysis, interpreted the data and critically reviewed and revised the manuscript. Barbara Busch contributed to the conception and design of the study and data acquisition, interpreted the data and reviewed and revised the manuscript critically. Peter Bartmann contributed to study design and conception, funding acquisition, interpreted the data and critically reviewed and revised the manuscript. Dieter Wolke conceived the study, obtained funding, designed the data collection, analysed and interpreted the data, and critically reviewed and revised the manuscript for important intellectual content. All authors approved the final manuscript as submitted and agree to be accountable for all aspects of the work in ensuring that questions related to the accuracy or integrity of any part of the work are appropriately investigated and resolved.

## FUNDING INFORMATION

Data collection for this study was supported by grants from the German Federal Ministry of Education and Science (BMBF) PKE24, JUG14, 01EP9504 and 01ER0801 and grant 733280 from the European Commission Horizon 2020 as part of the Research on European Children and Adults born Preterm (RECAP) Consortium. Elif Gonen is supported by the Study Abroad Program of The Republic of Türkiye Ministry of National Education. Dr. E Sabrina Twilhaar is supported by MSCA Postdoctoral Fellowship guarantee funding by UKRI's EPSRC (EP/X02105X/1). Dr. Nicole Baumann is supported by a Marie Sklodowska Curie Global Fellowship grant (No 886127). Prof Dieter Wolke, Dr. Barbara Busch and Prof Peter Bartmann are supported by a UKRI Frontier Research grant (EP/X023206/1) under the UK government's funding guarantee for ERC‐ADG grants.

## CONFLICT OF INTEREST STATEMENT

The authors have no conflict of interests relevant to this article to disclose.

## Supporting information


Data S1.

Data S2.

Data S3.

Table S1.

Table S2.

Table S3.

## Data Availability

Research data is not publicly available.
