# Supplementary material for: Changes in social relationships from 26 to 34 years of age in adults born very preterm
Source: Paediatr Perinat Epidemiol. 2024 Oct 27;39(1):15–26. doi: 10.1111/ppe.13133 (PMC11781515; doi:10.1111/ppe.13133)
Supplement: Supplementary file 1 — Data S1. [file PPE-39-15-s001.docx]

**Supplementary Document S1: Structurally Missing Data**

Structurally missing data (SMD) is defined as missing due to a sensible explanation since the information at baseline does not exist.^1,2^ For instance, individuals who had never been in a romantic relationship before could not answer the questions evaluating violence in romantic relationships. At 26- and 34-year assessments, fifty-one interviews, in total, had SMD in individual items (<5.3% missing values in each item) due to the lack of initial experience to answer the relevant question. Of these interviews, 24 were proxy and 8 were accompanied assessments.^a^ SMD was associated with the health status of the participants (e.g., severe disability).

As removing these interviews including SMD from the analyses, as recommended,^1^ may cause a selection bias and reduce the representativeness of the study, a conservative approach was chosen to handle this missing data. With this approach, if a certain situation experienced by the participant sensibly suggested an adverse effect on the relevant item, the missing data was assigned a score of 1 (non-optimal). Otherwise, the items were recoded as score 0 (optimal), which was the same as leaving them as missing. For example, for those who had no friends, the individual missing values for the item ‘No exchange of thoughts and feelings with friends’ were assigned a score of 1 (non-optimal), as there were no friends to share their thoughts and feelings with. After recoding each missing value as either score 0 or score 1 with this approach, linear mixed model analysis was conducted for the main analysis.

To evaluate the impact of our approach on the findings, the sum scores of each domain were recalculated without recoding SMD (i.e., leaving them as missing), and a sensitivity analysis was conducted. The results of the main and sensitivity analyses have been presented in Table 2.

**References**

1. Petrazzini BO, Naya H, Lopez-Bello F, Vazquez G, Spangenberg L. Evaluation of different approaches for missing data imputation on features associated to genomic data. *BioData min*. 2021;14:1-13. https://doi.org/10.1186/s13040-021-00274-7
2. Tripathi, Ashok Kumar, Geetanjali Rathee, and Hemraj Saini. "Taxonomy of missing data along with their handling methods." *2019 Fifth International Conference on Image Information Processing (ICIIP)*. IEEE, 2019.
