# Supplementary material for: Changes in social relationships from 26 to 34 years of age in adults born very preterm
Source: Paediatr Perinat Epidemiol. 2024 Oct 27;39(1):15–26. doi: 10.1111/ppe.13133 (PMC11781515; doi:10.1111/ppe.13133)
Supplement: Supplementary file 2 — Data S2. [file PPE-39-15-s005.docx]

**Supplementary Document S2.: Descriptive Variables**

**Neonatal Variables**

Gestational age was determined from maternal reports of the last menstrual period and serial ultrasounds during pregnancy. When the estimates of these two methods differed by more than 2 weeks, postnatal Dubowitz examination results were used.^1^ Birth weight and sex were documented in the birth records.^2^ Infants were classified as small for gestational age (SGA) if they weighed less than the sex-specific 10th percentile for their gestational age.^3^ A diagnosis of bronchopulmonary dysplasia (BPD) was assessed using the criterion of needing oxygen for at least 28 days and an X-ray diagnosis. Intraventricular hemorrhage (IVH) was assessed with ultrasound examination, graded into four categories according to Papile^4^ and subsequently coded into (0) no IVH or IVH grade 1 to 2 and (1) IVH grade 3 to 4.^5^

**Family socioeconomic status (SES)**

Family SES data were obtained by standard interviews with the infants’ parents during the first 10 days of life.^6^ SES was computed as a weighted composite score of the maternal highest educational qualification, paternal highest educational qualification, and occupation of the head of the family, and was grouped as low, middle, or high.^3, 7^

**Cognitive Assessment and Neuro-sensory Impairments**

Children’s intelligence was assessed with the German version of the Kaufman Assessment Battery for Children Mental Processing Composite (MPC) at 6 and 8 years.^8^ The Mental Processing Composite is the total of the Sequential (three subtests) and Simultaneous (five subtests) Processing scales.^9^ Reliability has been reported as good (0.83–0.98, split-half method), and construct validity as high (e.g. correlation of 0.70 with the Wechsler Intelligence Scale for Children-Revised total score). All cognitive assessments were performed by trained assistant psychologists who were blind to the birth status of the child.^10^

At ages 6 and 8 years, an Index of Neuro-sensory Impairment (NSI) was generated. Children were considered to have NSI if they had one or more of the following impairments: severe cerebral palsy [CP grade 3 or 4],^11^ hearing loss (not corrected), blindness, and IQ (KABC MPC Score) more than 2 SD below the mean.^6, 12^

**References**

1. Dubowitz LM, V D, D G. Clinical assessment of gestational age in the newborn infant*. J Pediatr*. 1970;77:1-10. doi: 10.1016/s0022-3476(70)80038-5.

2. Jaekel J, Wolke D, Bartmann P. Poor attention rather than hyperactivity/impulsivity predicts academic achievement in very preterm and full-term adolescents. *Psychol Med*. 2013;43(1):183-96. doi: 10.1017/S0033291712001031.

3. Eves R, Mendonça M, Bartmann P, Wolke D. Small for gestational age—cognitive performance from infancy to adulthood: an observational study. *BJOG*. 2020;127(13):1598-606. doi: 10.1111/1471-0528.16341.

4. Papile LA, Burstein J, Burstein R, Koffler H. Incidence and evolution of subependymal and intraventricular hemorrhage: a study of infants with birth weights less than 1,500 gm. *J Pediatr*. 1978;92(4): 529-34. doi: 10.1016/s0022-3476(78)80282-0.

5. Breeman LD, Jaekel J, Baumann N, Bartmann P, Wolke D. Neonatal predictors of cognitive ability in adults born very preterm: a prospective cohort study. *Dev Med Child Neurol*. 2017;59(5):477-83. doi: 10.1111/dmcn.13380.

6. Wolke D, Meyer R. Cognitive status, language attainment, and prereading skills of 6-year-old very preterm children and their peers: the Bavarian Longitudinal Study. *Dev Med* *Child Neurol*. 1999;41(2):94-109. doi: 10.1017/s0012162299000201.

7. Bauer A. *Ein Verfahren zur Messung des für das Bildungsverhalten relevanten Status (BRSS*). Frankfurt, Germany: Deutsches Institut für Internationale Pädagogische Forschung. 1988. [A procedure for the measurement of social status related to educational behaviour (BRSS)].

8. Melchers P, Preuss U. *K-ABC: Kaufman Battery for Children: Deutschsprachige Fassung.* Frankfurt, AM: Swets & Zeitlinger. 1991.

9. Kaufman A, Kaufman N. *Kaufman assessment battery for children: Interpretive manual.* Cirde Pines, MN: American Guidance Service. 1983.

10. Jaekel J, Wolke D, Chernova J. Mother and child behaviour in very preterm and term dyads at 6 and 8 years*. Dev Med Child Neurol.* 2012;54(8):716-23. doi: 10.1111/j.1469-8749.2012.04323.x.

11. Hagberg B, Hagberg G, Olow I, von Wendt L. The changing panorama of cerebral palsy in Sweden. V. The birth year period 1979-82. *Acta Paediatr Scand*. 1989;78(2):283–90. doi: 10.1111/j.1651-2227.1989.tb11071.x.

12. Jaekel J, Pluess M, Belsky J, Wolke D. Effects of maternal sensitivity on low birth weight children's academic achievement: a test of differential susceptibility versus diathesis stress. *J Child Psychol Psychiatry*. 2015;56(6):693-701. doi: 10.1111/jcpp.12331.
