# Supplementary material for: Changes in social relationships from 26 to 34 years of age in adults born very preterm
Source: Paediatr Perinat Epidemiol. 2024 Oct 27;39(1):15–26. doi: 10.1111/ppe.13133 (PMC11781515; doi:10.1111/ppe.13133)
Supplement: Supplementary file 3 — Data S3. [file PPE-39-15-s002.docx]

**Supplementary Document S3: Likelihood Ratio Test Results**

Linear mixed model (LMM) analysis was used to test each main and interaction effect in domains (overall social, parent, partner, and peers) from 26 to 34 years (within-subject factor). A random intercept was included in all models to adjust for repeated measurements within individuals. Random slopes were considered to allow the change in social relationship scores from 26 to 34 years to vary between individuals. A likelihood ratio test (LRT) was conducted for each domain to examine whether the inclusion of random slopes in addition to the random intercept provided a better model fit, using the degrees of freedom and corresponding critical chi-square values.^1^ LRT results provided insight regarding whether all individuals are assumed to follow the same trajectory or whether trajectories are assumed to vary from person to person.

According to the LRT results, random slopes in addition to the random intercept provided a better model fit for overall social relationships and parent relationships, but not for the partner and peer relationships. This indicates that the change in overall social relationships and parent relationships from 26 to 34 years varied between individuals.

In addition to the main analyses, three sensitivity analyses were conducted by repeating the LMM analysis. The LRT was also repeated for these analyses and its results are as follows.

- **Sensitivity Analyses 1: Structurally Missing Data**

A sensitivity analysis was conducted without recoding structurally missing data from 51 assessments. According to LRT results, and in line with the main analyses, random slopes in addition to random intercepts were included in the model for overall social relationships, but not for the partner and peer relationship domains. Different from the main analysis, fixed slopes were used for parent relationships, indicating that our conservative approach resulted in a more similar change over the years in parent relationships compared to the main analysis.

- **Sensitivity Analysis 2: Excluding Participants with Neurosensory Impairments**

A second sensitivity analysis was conducted by excluding participants with at least one neurosensory impairment. According to the test results, the change in social relationships of participants without any NSI varied in all domains instead of following a similar change pattern.

- **Sensitivity Analysis 3: IPCW**

A third sensitivity analysis was conducted by implementing inverse probability of censoring weighting (IPCW) before the main analysis. Based on the LRT results, and in line with the main analyses, random slopes in addition to random intercepts were included in the model for overall social relationship and parent relationship domains, but not for the partner and peer relationship domains. This shows that the weighted sample did not alter the change patterns within individuals in any domains.

Although the LRT results of these sensitivity analyses were different for some domains compared to the main analyses, the LMM analyses resulted in similar findings in all analyses.

**Reference**

1. Twisk JWR. *Applied longitudinal data analysis for medical science: A practical guide*. 3rd ed. Cambridge: Cambridge University Press; 2023.
https://doi.org/10.1017/9781009288002
