# Supplementary material for: Changes in social relationships from 26 to 34 years of age in adults born very preterm
Source: Paediatr Perinat Epidemiol. 2024 Oct 27;39(1):15–26. doi: 10.1111/ppe.13133 (PMC11781515; doi:10.1111/ppe.13133)
Supplement: Supplementary file 4 — Table S1. [file PPE-39-15-s006.docx]

**eTable S1.** Description of Indicators for Adult Social Relationship Characteristics

|  | | **26-year Assessment** | | | | | **34-year Assessment** | | | | | |
| --- | --- | --- | --- | --- | --- | --- | --- | --- | --- | --- | --- | --- |
| **Indicators** | | **VP/VLBW (n=260)**  ***n* (%)** | | **Term-born**  **(n= 229)**  ***n* (%)** | | **OR (95% CI)** | **VP/VLBW (n=214)**  ***n* (%)** | | **Term-born (n=202)**  ***n* (%)** | | **OR (95% CI)** | |
| **Social relationship characteristics – parent relationships** |  | |  | |  | | |  | |  | |  |
| Left parental home because of problems with parents  No exchange of thoughts and feelings with parents  Parents are not supportive regarding professional advancements or careers  Parents could not be asked for help if in need of care  Difficulties with being told uncomfortable truths by parents  Stressful conflicts with parents | | 14 (5.4)  94 (36.2)  67 (25.8)  49 (19.1)  112 (43.1)  103 (39.6) | | 17 (7.4)  81 (35.4)  58 (25.3)  44 (19.2)  109 (47.6)  103 (45.0) | | 0.7 (0.3, 1.5)  1.0 (0.7, 1.5)  1.0 (0.7, 1.5)  1.0 (0.6, 1.6)  0.8 (0.6, 1.2)  0.8 (0.6, 1.2) | 5 (2.3)  124 (57.9)  137 (64.0)  84 (39.4)  139 (65.0)  28 (13.1) | | 13 (6.4)  138 (68.3)  134 (66.3)  94 (47.0)  130 (65.3)  27 (13.4) | | 0.3 (0.1, 0.99)  0.6 (0.4, 0.95)  0.9 (0.6, 1.4)  0.7 (0.5, 1.1)  1.0 (0.7, 1.5)  1.0 (0.6, 1.7) | |
| **Social relationship characteristics – partner relationships** | | | | | | | | | | | | |
| Never dated  Never had sexual intercourse  Never in a serious relationship  Partner had been very mean and shouting  Partner has beaten, pushed, or physically attacked  Partner forced sex  Partner forbids going out or opens private mail or text messages | | 49 (18.8)  69 (26.5)  72 (27.7)  57 (21.9)  11 (4.2)  3 (1.2)  33 (12.7) | | 1 (0.4)  2 (0.9)  8 (3.5)  65 (28.4)  18 (7.9)  7 (3.1)  54 (23.6) | | 52.9 (7.2, 386.8)  41.0 (9.9, 169.5)  10.6 (5.0, 22.5)  0.7 (0.5, 1.1)  0.5 (0.2, 1.1)  0.4 (0.1, 1.4)  0.5 (0.3, 0.8) | 32 (15.0)  44 (20.8)  46 (21.6)  32 (15.0)  12 (5.6)  6 (2.8)  26 (12.2) | | 1 (0.5)  0 (0.0)  3 (1.5)  36 (17.9)  13 (6.5)  0 (0.0)  26 (12.9) | | 35.5 (4.8, 262.7)  NA  18.3 (5.6, 59.8)  0.8 (0.5, 1.4)  0.9 (0.4, 1.9)  NA  0.9 (0.5, 1.7) | |
| **Social relationship characteristics – peer relationships** | | | | | | | | | | | | |
| No best friend after time at school  None to only two or three friends after time at school  No circle of friends after time at school  No exchange of thoughts and feelings with friends  Friends are not supportive regarding professional advancements or careers  Friends could not be asked for help if in need of care  Difficulties with being told uncomfortable truths by friends  Stressful conflicts with friends  Not involved in social activities (e.g. dancing, music, scouts) for more than a year after time at school  Never gone out to bars or clubs after time at school | | 67 (25.8)  72 (27.7)  112 (43.1)  135 (51.9)  219 (84.2)  233 (89.6)  161 (61.9)  32 (12.3)  162 (62.3)  39 (15.0) | | 48 (21.0)  34 (14.8)  66 (28.8)  88 (38.4)  176 (76.9)  200 (87.3)  119 (52.0)  37 (16.2)  117 (51.1)  4 (1.7) | | 1.3 (0.9, 2.0)  2.2 (1.4, 3.5)  1.9 (1.3, 2.7)  1.7 (1.2, 2.5)  1.6 (1.02, 2.5)  1.4 (0.8, 2.5)  1.5 (1.05, 2.2)  0.7 (0.4, 1.2)  1.6 (1.1, 2.3)  9.9 (3.5, 28.2) | 35 (16.4)  43 (20.1)  64 (29.9)  92 (43.0)  181 (84.6)  197 (92.5)  138 (64.5)  11 (5.2)  94 (43.9)  49 (22.9) | | 30 (14.9)  7 (3.5)  39 (19.3)  73 (36.1)  180 (89.1)  185 (92.5)  127 (63.8)  7 (3.5)  60 (29.7)  9 (4.5) | | 1.1 (0.7, 1.9)  7.0 (3.0, 16.0)  1.8 (1.1, 2.8)  1.3 (0.9, 2.0)  0.7 (0.4, 1.2)  1.0 (0.5, 2.1)  1.0 (0.7, 1.5)  1.5 (0.6, 4.0)  1.9 (1.2, 2.8)  6.4 (3.0, 13.4) | |

Abbreviation: OR, odds ratios; NA, not available
