# Supplementary material for: Changes in social relationships from 26 to 34 years of age in adults born very preterm
Source: Paediatr Perinat Epidemiol. 2024 Oct 27;39(1):15–26. doi: 10.1111/ppe.13133 (PMC11781515; doi:10.1111/ppe.13133)
Supplement: Supplementary file 5 — Table S2. [file PPE-39-15-s003.docx]

|  | | | **VP/VLBW** | | | **Term-born** | | | |
| --- | --- | --- | --- | --- | --- | --- | --- | --- | --- |
| **Descriptive Characteristics** | | | **Participants**  **(*n*= 262, 63.7%)** | **Lost to follow-up**  **(*n*=149, 36.3%)** | **Difference**  **(95% CI)** | **Participants**  **(*n*=230, 74.7%)** | | **Lost to follow-up**  **(*n*=78, 25.3%)** | **Difference**  **(95% CI)** |
| Birthweight, mean (SD), g | | | 1325 (316.6) | 1262 (287.6) | 63.3 (1.5, 125.1) | | 3361 (445.3) | 3448 (443.7) | -86.4 (-201.1, 28.3) |
| Gestational age, mean (SD), wk | | | 30.6 (2.2) | 30.4 (2.4) | 0.2 (-0.3, 0.6) | | 39.6 (1.2) | 39.7 (1.2) | -0.01 (-0.3, 0.3) |
| SGA (<10%), No. (%) | | | 109 (41.6) | 67 (45.0) | 0.9 (0.6, 1.3) | | 23 (10.0) | 8 (10.3) | 1.0 (0.4, 2.3) |
| Sex, N (%) | | |  |  | 1.1 (0.8, 1.7) | |  |  | 0.6 (0.4, 1.04) |
|  | Male | | 138 (52.7) | 74 (49.7) |  |  | 108 (47.0) | 46 (59.0) |  |
| Family SES at birth, No. (%) | | |  |  | 1.00 (Reference)  1.8 (1.2, 2.7)  0.5 (0.3, 0.8) | |  |  | 1.00 (Reference)  1.7 (0.97, 2.9)  0.3 (0.2, 0.5) |
|  | SES-high | | 55 (21.0) | 27 (18.1) |  |  | 77 (33.5) | 15 (19.2) |  |
|  | SES-middle | | 123 (46.9) | 50 (33.6) |  |  | 98 (42.6) | 24 (30.8) |  |
|  | SES-low | | 84 (32.1) | 72 (48.3) |  |  | 55 (23.9) | 39 (50.0) |  |
| Bronchopulmonary dysplasia, No. (%) | | | 137 (52.3) | 79 (53.0) | 1.0 (0.6, 1.5) | | NA | NA | NA |
| Intraventricular haemorrhage, No. (%) | | |  |  |  | |  |  |  |
|  | | Stage 3 or 4 | 20 (7.6) | 10 (6.7) | 1.1 (0.5, 2.5) | | 0 (0.0) | 0 (0.0) | NA |
| Neurosensory impairments (NSI), No. (%) | | |  |  |  | |  |  |  |
|  | One or more imp. | | 42 (16.0) | 32 (21.5) | 0.7 (0.4, 1.2) | | 1 (0.4) | 0 (0.0) | NA |

**eTable S2.** Descriptive Characteristics of Eligible VP/VLBW and Term-born Adults

Note. Independent samples t-test for continuous variables, and $X^{2}$test or Fisher’s exact test for categorical variables were performed. Mean differences for continuous variables and odds ratios for categorical variables were reported, respectively.

Abbreviation: SD, standard deviation; SGA, small for gestational age; SES, socioeconomic status; NA, not available
